# CD206^+^ tumor-associated macrophages interact with CD4^+^ tumor-infiltrating lymphocytes and predict adverse patient outcome in human laryngeal squamous cell carcinoma

**DOI:** 10.1186/s12967-023-03910-4

**Published:** 2023-03-03

**Authors:** Yu Heng, Xiaoke Zhu, Hanqing Lin, Ma jingyu, Xuping Ding, Lei Tao, Liming Lu

**Affiliations:** 1 ENT Institute and Department of Otorhinolaryngology, Eye & ENT Hospital, Fudan University, Shanghai, 200031 People’s Republic of China; 2 Shanghai Institute of Immunology, Department of Immunology and Microbiology, Shanghai Jiao Tong University School of Medicine, Shanghai, 200025 People’s Republic of China

**Keywords:** Tumor-associated macrophages, Laryngeal squamous cell carcinoma, Prognosis, Macrophage infiltrating pattern, CD4^+^ T cell interaction, Immunosuppressive microenvironment

## Abstract

**Background:**

Tumor-associated macrophages (TAMs) are major component in the tumor microenvironment (TME) and play regulatory role in tumor progression. We aimed to investigate the infiltration and prognostic value of TAMs in laryngeal squamous cell carcinoma (LSCC) and to reveal the underlying mechanism of TAM subgroups in tumorigenesis.

**Methods:**

Hematoxylin and eosin (HE) staining were performed to define the tumor nest and stroma of LSCC tissue microarrays. CD206 + /CD163 + and iNOS + TAM infiltrating profiles were obtained and analyzed through double-labeling immunofluorescence and immunohistochemical staining. The recurrence-free (RFS) and overall survival (OS) curves based on the infiltration of TAMs were plotted using the Kaplan-Meier method. Infiltration of macrophages, T lymphocytes and their corresponding subgroups were analyzed in fresh LSCC tissue samples by flow cytometry.

**Results:**

We found that CD206^+^ rather than CD163^+^ M2-like TAMs were the most enriched population in the TME of human LSCC. CD206^+^ macrophages localized mostly in the tumor stroma (TS) rather than the tumor nest (TN) region. In contrast, relatively low infiltration of iNOS^+^ M1-like TAMs were found in the TS and almost none in the TN region. High level of TS CD206^+^ TAM infiltration correlated with poor prognosis. Interestingly, we identified a HLA-DR^high^ CD206^+^ macrophage subgroup that was significantly associated with the tumor-infiltrating CD4^+^ T lymphocytes and showed different surface costimulatory molecule expression than that of the HLA-DR^low/^-CD206^+^ subgroup. Taken together, our results indicate that HLA-DR^high^-CD206^+^ is a highly activated subgroup of CD206 + TAMs that may interact with CD4 + T cells through MHC-II axis and promote tumorigenesis.

**Supplementary Information:**

The online version contains supplementary material available at 10.1186/s12967-023-03910-4.

## Introduction

Laryngeal squamous cell carcinoma (LSCC) accounts for over one-quarter of all head and neck carcinoma [1]. Though traditional treatments of surgery, radiotherapy, and chemotherapy have decreased the overall incident rate of LSCC, the long-term survival rate has dropped from 66 to 63% over the past 40 years [2, 3]. To overcome the bottleneck of existing treatments, immunotherapy has become an emerging therapeutic strategy. One of the research focuses in tumor immunotherapy is to investigate the interactions of tumor cells, immune cells and stroma, which may provide new directions to reverse the immunosuppressive tumor microenvironment (TME) to facilitate immunotherapy [4, 5].

Macrophages are innate myeloid cells traditionally divided into two categories: M1-polarized macrophages (M1 macrophages) and M2-polarized macrophages (M2 macrophages) which are promoted by Th1- and Th2-derived cytokines, respectively [6, 7]. Tumor-associated macrophages (TAMs) are one of the most abundant immune cells in the TME. Though TAMs are generally known to associate with poor prognosis in many types of solid tumor and play key roles in tumorigenesis, angiogenesis [8, 9], and metastatic progression [10], their functions cannot be simply explained by the M1 or M2 phenotype. For example, in some cases, the M2-like macrophages correlate with unsatisfactory survival outcome, possibly by causing abnormal Th2 immune response. However, other studies have also reported that high macrophage infiltration leads to better survival [11–15]. Another example is CD68 + , a pan macrophage surface marker, which associates with both adverse and satisfactory prognosis in different types of cancer [17]. In addition, the TME also plays essential role in regulating the function of TAMs and the cognate interaction between tumor-infiltrating CD4 + T-helper type 1 (Th1) cells and TAMs may shift the TAMs toward M1-like activity [20]. However, up to now, the interaction between TAMs and tumor-infiltrating T lymphocytes are still largely elusive in LSCC.

In this study, we dissected TAMs in LSCC tumor into more specified subtypes and evaluated the correlation of each sub-group with the recurrence and survival outcomes of LSCC patients. We focused on the clinicopathologic and prognostic significance of CD68 + /iNOS + M1-like, and CD68 + /CD206 + M2-like TAMs by examine their compositions in FFPE human primary LSCC lesions. We also identified a subgroup of HLA-DR^high^ CD206^+^ TAM that may interact with tumor-infiltrating CD4 + T and may be a potential therapeutic target for LSCC.

## Materials and methods

### Ethical approval

The study was performed according to the principles of the Declaration of Helsinki, and the Medical Research Council of the Eye, Ear, Nose and Throat Hospital, Fudan University, Shanghai, China approved this study (No. KJ2008-01). All patients gave informed consent to take part in the study.

### Study population, collected information, and tissue microarray (TMA) construction

Human LSCC tissue microarrays (Wuhan Biosci Biotechnology Co.; Ltd, Wuhan, China) were serially sliced and contained 80 pairs of tissue specimens including cancer tissue and adjacent normal tissues from patients diagnosed as LSCC and received surgical treatments without radiotherapy or chemotherapy therapy (both before and after surgery) between June 2014 and September 2017 at our medical center. Each of the TMA sections consisted of 4-µm-thick tissues. Hematoxylin and eosin (HE) staining were performed and the definitions of tumor nest and tumor stroma areas were confirmed by two board-certified pathologists. The fresh tumor tissues of LSCC of 15 patients receiving surgical treatments were researched using flow cytometry for detecting tumor-infiltrating T lymphocytes and TAMs. Patients’ medical records were reviewed to acquire their baseline clinical and disease characteristics, including sex, age, smoking history, alcohol drinking, grade of differentiation, T stage, N stage, and tumor diameter. The classification of the disease for all patients was determined according to the Eighth Edition of the American Joint Committee on Cancer (AJCC) Tumor, Node, Metastasis (TNM) guidelines. Overall survival (OS) and recurrence-free survival (RFS) were regarded as the end events of this study; data on time to recurrence and death were obtained during the follow-up.

### Double-labeling immunofluorescence (IF) and IF staining reagents used and antibody dilution

The antibodies involving in our study were diluted as follows: Anti-CD68 antibody (76437, clone D4B9C, CST), 1:200; Anti-MMR/CD206 antibody (AF2534, R&D Systems), 1:100; Anti-iNOS antibody (610328, BD Biosciences), 1:100; Anti-CD163 antibody (ab182422, Abcam), 1:100; DAPI (Solarbio#C0060), working fluid.

### Double-labeling immunofluorescence (IF)

TMA slices were deparaffinized and rehydrated, antigen retrieval and blocked according to the standard manner of immunohistochemical (IHC) protocol. The slices were then incubated with anti-CD68 primary antibody (1:200) and goat monoclonal anti-iNOS primary antibody (1:500, Abcam) or goat monoclonal anti-CD206 primary antibody (1:500, Abcam) overnight at 4 °C. After three washes of PBS of 10 min each, the secondary antibodies were conjugated with different fluorochromes and both sections were incubated in the dark for 60 min. After washing, the slides were mounted with Vectashield Hardset mounting medium with/without 4′,6-diamidino-2-phenylindole (DAPI; Solarbio, C0060).

### iNOS^+^ M1 and CD206^+^ M2 TAMs counting

Each cancer tissue was evaluated and divided into two regions: tumor nest region and tumor stroma region by two board-certified pathologists according to HE staining (Additional file 1: Fig. S1). Under the microscope with 10 × 40 magnification, three fields of vision with the highest densities of iNOS + M1 and CD206 + M2 TAMs in tumor nest, tumor stroma, and adjacent normal tissue regions were selected to count M1 and M2 TAMs, respectively. As a result, the average number of the selected three regions was recorded as the number of M1 and M2 TAMs for each specimen.

### Immunohistochemical staining

4–5 μm thick sections were cut from formalin-fixed, para-embedded tissue. Sections were then deparaffinized using dimethylbenzene for 10 min, followed by rehydration in a series of graded alcohol (100%, 90%, 85%, 80%, 75%, 60%, 50%, 30%, 5 min for each concentration). Antigens were then retrieved by heating for 15 min using a microwave oven. After cooling to room temperature, the section was placed in tris buffered saline Tween buffer for 6 min. Following antigen retrieval, slides were blocked and labeled with primary antibodies.

### Human LSCC samples and preparation of single-cell suspensions

Fresh tumor tissues of LSCC patients were collected during standard surgical procedures from the Department of Head and Neck Surgery, Eye & ENT Hospital of Fudan University. Tumor tissues not needed for diagnostic purposes were minced and subjected to enzymatic digestion for 2 h with collagenase IV at 1.2 mg/ml (C5138, Sigma) in RPMI-1640 medium at 37 °C. The samples were then filtered through a 70-μm cell strainer (352350, Corning Falcon), and washed with phosphate-buffered saline.

### Flow-cytometric analysis

Cell suspensions were washed in staining buffer and stained with fluorochrome-conjugated monoclonal antibodies for cell surface markers to identify macrophage and T lymphocytes and their subgroups. Zombie UV Fixable Viability kit (423107. BioLegend) was used for assessing live status. Cells were first incubated with the live/dead dye for 20 min at room temperature, then was washed and stained for cell surface markers for 30 min at room temperature in the dark. Foxp3 / Transcription Factor Staining Buffer Set (00-5523, Invitrogen) was used for performing intracellular staining. All the antibodies used were listed in Additional file 5: Table S1. The gating strategies for tumor-associated macrophages and tumor-infiltrating T lymphocytes and their respective subgroups were shown in Additional file 2: Fig. S2. Data were acquired using an LSR Fortessa (BD Biosciences) and analyzed with FlowJo software version 10.

### Statistical analysis

Clinical data are shown in numbers or percentages according to relevant clinicopathology. Chi-square and independent t-test were used to make comparisons between categorical and continuous variables, respectively. Overall survival (OS) was calculated from the surgery date until the date of death or the date of the last follow-up, while recurrence-free survival (RFS) was defined as the survival time from surgery to tumor recurrence. OS and RFS curves were determined using the Kaplan-Meier method and compared using the log-rank test. All statistical analyses were conducted using SPSS 24.0 software (SPSS Inc., Chicago, IL, USA), and a p-value < 0.05 was considered as statistically significant.

## Results

### CD206^+^ macrophage is the major TAMs in LSCC tumors

In LSCC tumor, the interaction of a nest of tumor cells with adjacent stroma cells is critical for tumor progression. To visualize the localization of different types of TAMs in LSCC tumor, we analyzed the FFPE sample of 80 LSCC patients who received only surgical treatment without pre- and post-operative adjuvant therapies. The demographic and clinicopathological characteristics of the patients are summarized in Table 1. The median age at diagnosis was 62.6 years (ranging from 46.0 to 80.0), and 78/80 (97.5%) of the patients were men. Fifty-six patients (70.0%) had a history of smoking while 43 (53.7%) had a history of alcohol drinking. According to the 8^th^ Edition of the AJCC guidelines, 7 (8.8%) patients had stage I disease, 6 (7.5%) patients stage II, 32 (40.0%) patients stage III, and 35 (43.8%) patients stage IV.

**Table 1:** Demographic and clinicopathological characteristics of the patients with LSCC (tissue microarray of 80 patients)

Characteristics	All patients (N = 80)	
	No	%
Age (mean ± SD)	62.6 ± 7.5	
< 65	46	57.5
≥ 65	34	42.5
Gender		
Male	78	97.5
Female	2	2.5
Smoking history		
No	24	30.0
Yes	56	70.0
Alcohol drinking		
No	37	46.3
Yes	43	53.7
Primary site		
Glottic	49	61.2
Supraglottic	31	38.8
T stage		
T1	7	8.8
T2	11	13.8
T3	36	45.0
T4	26	32.5
N stage		
N0	48	60.0
N1	18	22.5
N2	14	17.5
N3	0	0.0
Pathological stage		
Stage I	7	8.8
Stage II	6	7.5
Stage III	32	40.0
Stage IV	35	43.8
Tumor differentiation		
Well or moderately	71	88.8
Poorly	9	11.2
Tumor nest iNOS + M1 infiltration		
No	80	100.0
Yes	0	0.0
Tumor nest CD206 + M2 infiltration		
No	52	65.0
Yes	28	35.0
Tumor stroma iNOS + M1 infiltration		
No	67	83.8
Yes	13	16.3
Tumor stroma CD206 + M2 infiltration		
No	27	33.7
Yes	53	66.3

**Fig. 1:**
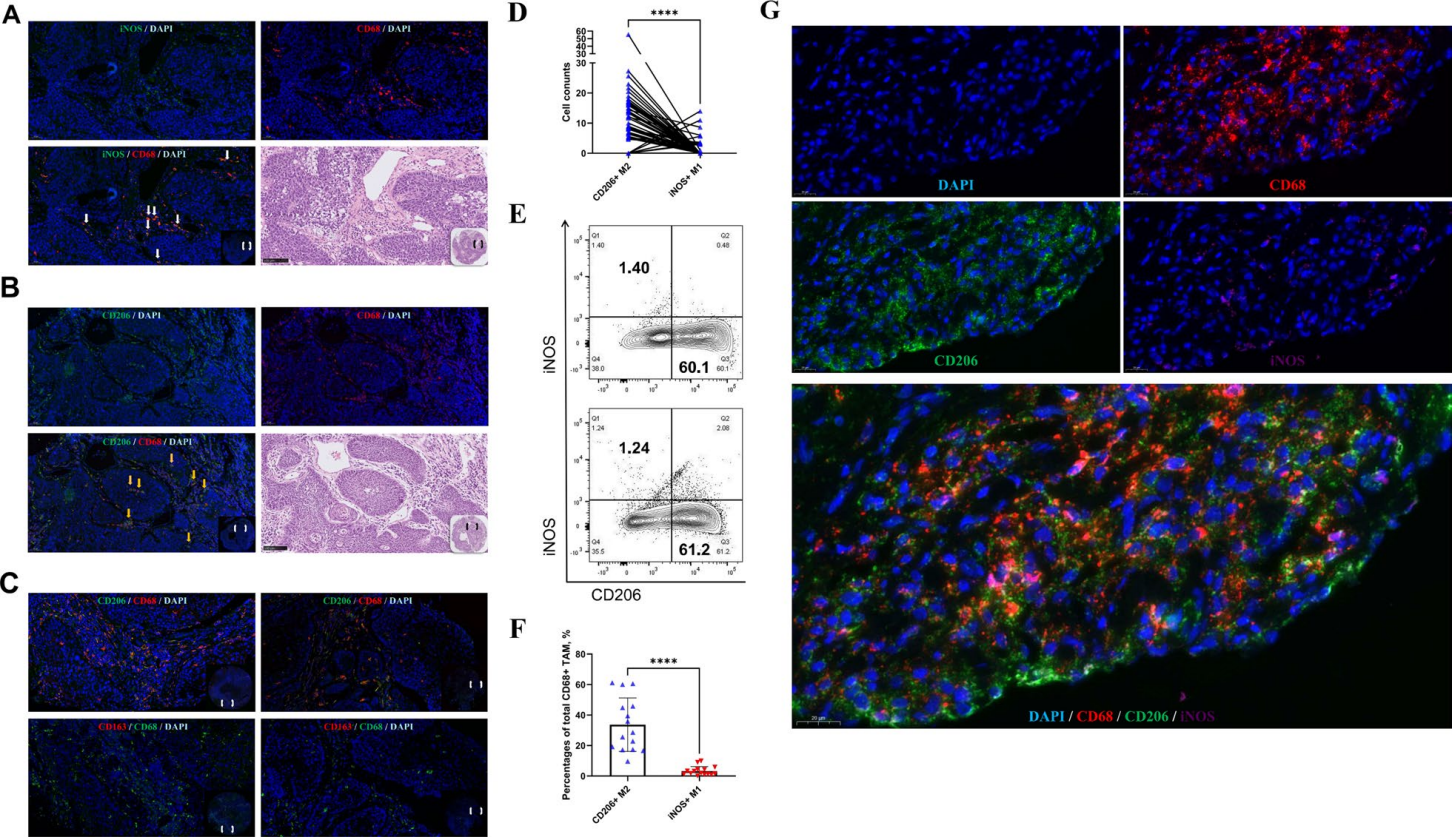
CD206^+^ M2-like macrophage was the dominnat TAM population in the tumor microenvironment of LSCC. **A** Immunofluorescence staining of CD68^+^iNOS^+^ M1-like TAMs on LSCC tumor sections. iNOS (green), CD68 (red) and DAPI (blue). The corresponding HE staining was shown in the lower right-hand panel. **B** and** C** Double-immunofluorescence staining of CD68^+^CD206^+^ and CD68^+^CD163^+^ M2-like TAMs. **B** CD206 (green) and CD68 (red) staining of LSCC sections. Total nuclei were co-stained with DAPI (blue); The corresponding HE staining was shown in the lower right hand panel. **C** Two representative double-immunofluorescence staining of CD68^+^CD206^+^ and CD68^+^CD163^+^ TAMs on LSCC tissues. Upper panels showed CD68^+^(red) and CD206^+^ (green) and lower panels showed CD68^+^ (green) and CD163^+^ (red). **D** The infiltrating level of CD68^+^CD206^+^ TAMs was significantly higher than that of CD68^+^iNOS^+^ in LSCC tissue, p-value < 0.0001; **E** Representative dot plots displayed CD206^+^ M2 and iNOS^+^ M1 in fresh tumor tissues of LSCC patients using flow-cytometric analysis. The corresponding statistics analysis was shown in **F** using Paired sample t test, with statistical significance indicated as follows: ****p < 0.0001; **G** Multiplex immunofluorescence staining of CD68, CD206 and iNOS of LSCC tissue

We first visualized the localization of three subtypes of TAMs using double-immunofluorescence staining of pan macrophage marker CD68 combined with iNOS (M1-like), CD163 (M2-like), and CD206 (M2-like), respectively, on patient tissue microarray (TMA). The results showed low level of iNOS^+^ macrophages infiltration in the tumor microenvironment of LSCC. iNOS^+^ TAMs was observed in only 16.3% (13/80) of LSCC tumors and all of them were detected in the tumor stroma (TS) (Fig. 1A), with no existence in tumor nest (TN) or adjacent normal tissue (ANT). On the other hand, CD206^+^ macrophages were more commonly detected in the TME (Fig. 1B) with 66.3% (53/80) in TS and 35.0% (28/80) in TN. Interestingly, a small amount of CD206^+^ macrophage were also found in the ANT region of 15 (18.8%) tumors. Surprisingly, we did not detect CD68^+^CD163^+^ macrophage in the serial sections of all 80 tumors, though CD163 was commonly expressed by macrophage (Fig. 1C). Statistically, paired t-test showed that the infiltration level of CD206^+^ macrophages was significantly higher than that of iNOS^+^ M1-like macrophages in patients with LSCC (p-value < 0.0001, Fig. 1D). This result suggested that CD206^+^ macrophage is the major M2-like TAMs in LSCC.

We further confirmed the enrichment of CD206^+^ macrophages in the TME by analyzing the composition of TAMs from fresh tumor tissue samples of LSCC patients by flow cytometry. Consistently, the infiltration of CD206 + TAMs was significantly higher than that of iNOS + TAMs (p-value < 0.0001, Fig. 1E, F). Therefore we concluded that CD206^+^ macrophage is the major TAMs in the TME of LSCC (Fig. 1G, Additional file 3: Fig. S3).

### Infiltration of CD206^+^ macrophages was higher in tumor stroma region compared to tumor nest

**Fig. 2:**
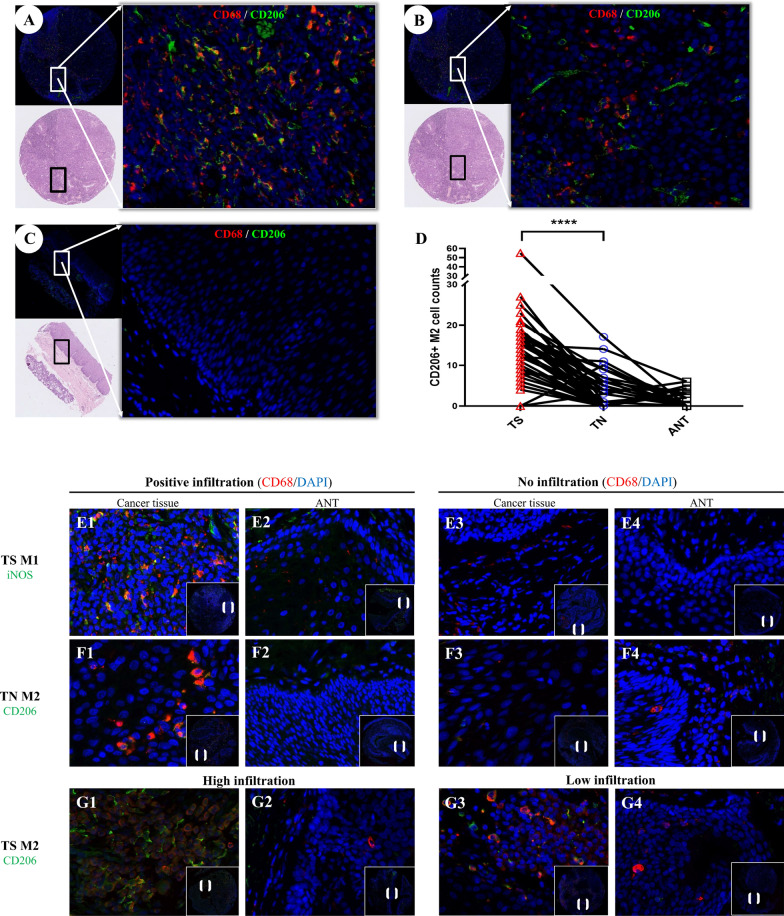
The infiltration pattern of CD68^+^CD206^+^ M2-like macrophages in the TME. **A**–**C** The infiltrating level of CD68 + CD206 + M2-like macrophages in tumor stroma **A**, tumor nest **B**, and corresponding adjacent normal tissue** C**. **D** Patients with LSCC exhibited different infiltrating levels of CD68^+^iNOS^+^ M1 and CD68^+^CD206^+^ M2 TAMs; Paired sample t test was used and statistical significance indicated as follows: ****p < 0.0001. **E** Immunofluorescent staining of patient tumor samples in cancer tissue and ANT region. Patients with positive **E1** and negative **E3** TS CD68^+^iNOS^+^ M1 TAM infiltration, and the CD68^+^iNOS^+^ M1 infiltration **E2**, **E4**; **F** Patients with positive **F1** and negative **F3** TN CD68^+^CD206^+^ M2 TAM infiltration, and the CD68^+^CD206^+^ M2 infiltration of their corresponding ANT **F2**, **F4**. **G** Patients with high **G1** and low **G3** levels of TS CD68^+^CD206^+^ M2 TAM infiltration, and the CD68^+^CD206^+^ M2 infiltration of their corresponding ANT **G2**, **G4**. *LSCC*, Laryngeal squamous cell carcinoma; *TN*, Tumor nest; *TS*, Tumor stroma; *ANT*, Adjacent normal tissue

We next examined the localization of CD206^+^ macrophages in more detail using double-immunofluorescence staining and found CD206^+^ macrophage infiltration in the TS region in 53 of the 80 LSCC tumors, ranging from 0 to 55.67 while only 28 tumors showed infiltration in the TN region, ranging from 0 to 17.0. Paired t-test confirmed that the infiltration of CD206^+^ macrophages was significantly higher in the TS regions than in the TN regions (p-value < 0.0001, Fig. 2A–D). Moreover, we could barely detect CD206^+^ macrophages in the ANT regions, with only 15 tumors showed positive CD206^+^ infiltration and all less than 6.0.

### Correlation between iNOS^+^ and CD206^+^ TAMs infiltration and the clinicopathological characteristics of LSCC

To evaluated the correlation between TAMs and clinicopathological characteristics of LSCC tumors, we stratified LSCC patients by TS infiltrated CD206^+^ TAMs. Since the median number of TS CD206^+^ TAMs was 8.0 for all patients and 14.0 for patients with positive TS infiltration, we defined patients with at least 14.0 TS infiltration value of TS CD206^+^ TAMs as high infiltration group, and those with value less than 14.0 as low infiltration group (Fig. 2E–G). In addition, each group was further divided into positive-infiltration or no infiltration groups based on the total presence of TS iNOS^+^ and TN CD206^+^ TAMs (Fig. 4).

We then analyzed the correlation between the infiltration of TS iNOS^+^ M1-like macrophages, TS and TN M2-like macrophages and the clinicopathological features and prognosis of LSCC patients (Table 2). The results indicated that patients with TS iNOS^+^ TAMs infiltration showed a significantly lower probability of tumor recurrence in the long-term follow-up compared to those with no TS iNOS^+^ TAMs infiltration (p-value = 0.037). On the contrary, TN CD206^+^ TAMs infiltrations was significantly correlated with worse recurrence and long-term survival outcome (both p-value < 0.001), and a similar correlation was found for TS CD206^+^ TAMs (p-value = 0.016 and 0.034, respectively). Interestingly, among the 53 patients with positive TS CD206^+^ TAMs infiltration, those with higher CD206^+^ TAMs infiltration exhibited unsatisfactory recurrence and survival outcomes compared to those with low TS CD206^+^ infiltration (p-value < 0.001, respectively). In addition, patients with pathological stage IV disease were more likely to show positive TS CD206^+^ infiltration than those with stage I-III disease (80.0% (28 in 35) vs 55.6% (25 in 45), p-value = 0.022). Among all patients that exhibited positive TS CD206^+^ infiltration, patients with stage IV disease showed comparable infiltrating levels to those with stage I-III disease (high level of TS CD206^+^ TAMs rate: 53.6% (15 in 28) and 48.0% (12 in 25) for stage IV and stage I-III, respectively, p-value = 0.685).

**Table 2:** Correlations between the M1 and M2 TAMs infiltration levels and the clinicopathologic characteristics of LSCC (intra-groups analysis)

Features	Cases	TS iNOS + M1 TAM count		p-value	TN CD206 + M2 TAM count		p-value	TS CD206 + M2 TAM count		p-value	TS CD206 + M2 TAM count		p-value
		Yes	No		Yes	No		Yes	No		High	Low	
		N = 13 (N,%)	N = 67 (N,%)		N = 28 (N,%)	N = 52 (N,%)		N = 53 (N,%)	N = 27 (N,%)		N = 27 (N,%)	N = 26 (N,%)	
Age				0.366			0.602			0.466			0.016
< 65	46	6 (13.0)	40 (87.0)		15 (32.6)	31 (67.4)		32 (69.6)	14 (30.4)		12 (37.5)	20 (62.5)	
≥ 65	34	7 (20.6)	27 (79.4)		13 (38.2)	21 (61.8)		21 (61.8)	13 (38.2)		15 (71.4)	6 (28.6)	
Smoking history				0.209			0.082			0.135			0.094
No	24	2 (8.3)	22 (91.7)		5 (20.8)	19 (79.2)		13 (54.2)	11 (45.8)		4 (30.8)	9 (69.2)	
Yes	56	11 (19.6)	45 (80.4)		23 (41.1)	33 (58.9)		40 (71.4)	16 (28.6)		23 (57.5)	17 (42.5)	
Alcohol drinking				0.221			0.981			0.808			0.328
No	37	4 (10.8)	33 (89.2)		13 (35.1)	24 (64.9)		24 (64.9)	13 (35.1)		14 (58.3)	10 (41.7)	
Yes	43	9 (20.9)	34 (79.1)		15 (34.9)	28 (65.1)		29 (67.4)	14 (32.6)		13 (44.8)	16 (55.2)	
Primary site				0.222			0.683			0.822			0.865
Glottic	49	6 (12.2)	43 (87.8)		18 (36.7)	31 (63.3)		32 (65.3)	17 (34.7)		16 (50.0)	16 (50.0)	
Supraglottic	31	7 (22.6)	24 (77.4)		10 (32.3)	21 (67.7)		21 (67.7)	10 (32.3)		11 (52.4)	10 (47.6)	
T stage				0.502			0.466			0.098			0.761
T12	18	2 (11.1)	16 (88.9)		5 (27.8)	13 (72.2)		9 (50.0)	9 (50.0)		5 (55.6)	4 (44.4)	
T34	62	11 (17.7)	51 (82.3)		23 (37.1)	39 (62.9)		44 (71.0)	18 (29.0)		22 (50.0)	22 (50.0)	
N stage				0.083			0.293			0.562			0.646
N0	48	5 (10.4)	43 (89.6)		19 (39.6)	29 (60.4)		33 (68.8)	15 (31.2)		16 (48.5)	17 (51.5)	
N +	32	8 (25.0)	24 (75.0)		9 (28.1)	23 (71.9)		20 (62.5)	12 (37.5)		11 (55.0)	9 (45.0)	
Pathological stage				0.423			0.723			0.022			0.685
Stage I-III	45	6 (13.3)	39 (86.7)		15 (33.3)	29 (66.7)		25 (55.6)	20 (44.4)		12 (48.0)	13 (52.0)	
Stage IV	35	7 (20.0)	28 (80.0)		13 (37.1)	23 (62.9)		28 (80.0)	7 (20.0)		15 (53.6)	13 (46.4)	
Tumor differentiation				0.606			0.911			0.978			0.36
Well/moderately	71	11 (15.5)	60 (84.5)		25 (35.2)	29 (64.8)		47 (66.2)	24 (33.8)		25 (53.2)	22 (46.8)	
Poorly	9	2 (22.2)	7 (77.8)		3 (33.3)	6 (66.7)		6 (66.7)	3 (33.3)		2 (33.3)	4 (66.7)	
Recurrence				0.037			<0.001			0.016			0.000
No	54	12 (22.2)	42 (77.8)		10 (18.5)	44 (81.5)		31 (57.4)	23 (42.6)		8 (25.8)	23 (74.2)	
Yes	26	1 (3.8)	25 (96.2)		18 (69.2)	8 (30.8)		22 (84.6)	4 (15.4)		19 (86.4)	3 (13.6)	
Prognosis				0.055			<0.001			0.013			0.000
Live	56	12 (21.1)	44 (78.9)		12 (21.1)	44 (78.9)		33 (57.9)	23 (42.1)		9 (27.3)	24 (72.7)	
Died	24	1 (4.3)	23 (95.4)		16 (69.6)	8 (30.4)		20 (87.0)	4 (13.0)		18 (90.0)	2 (10.0)	

### Prognostic value of iNOS^+^ and CD206^+^ TAM infiltration in LSCC patients

**Fig. 3:**
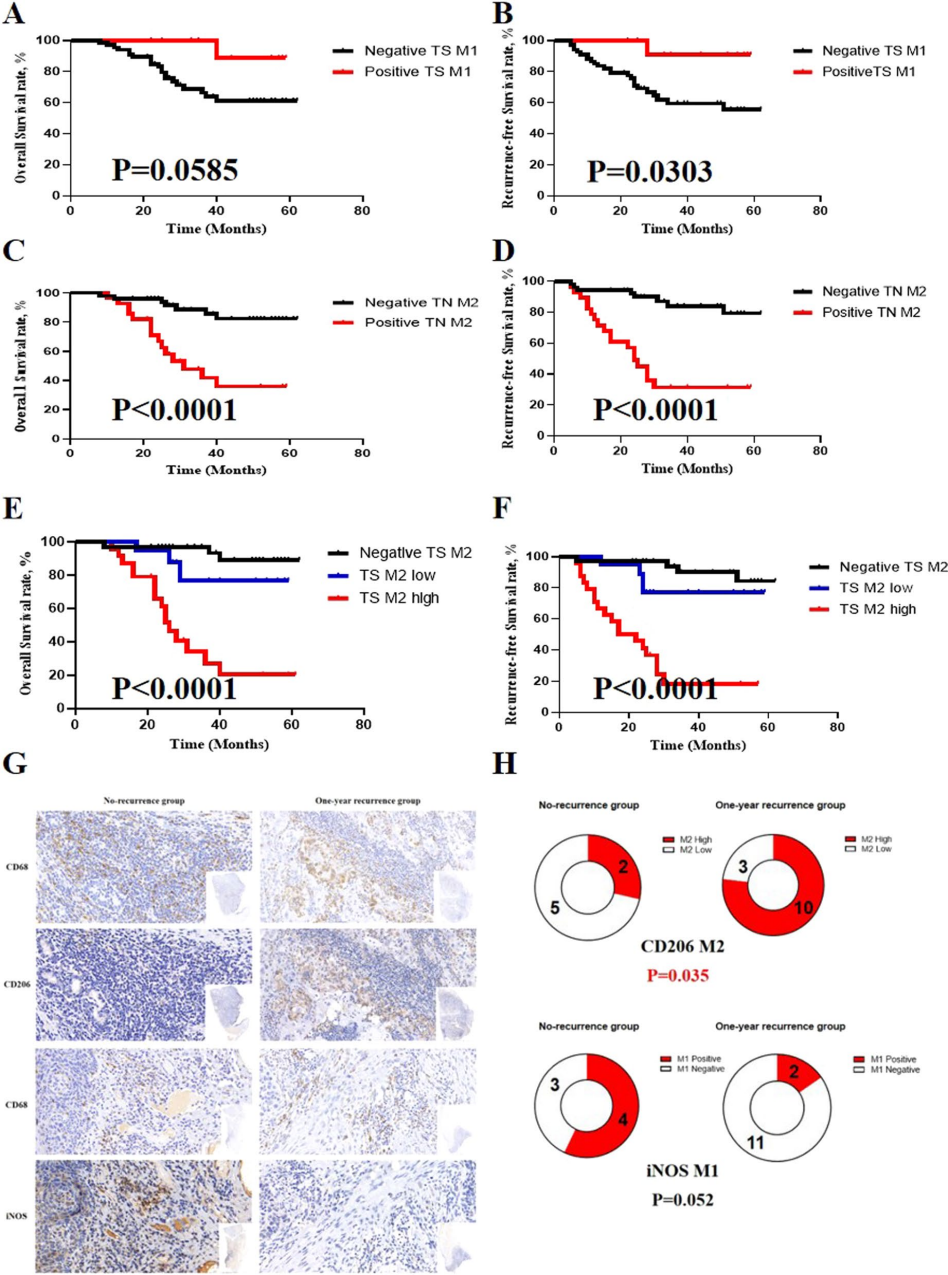
Kaplan–Meier curves for OS and RFS of LSCC cohort (n = 80). **A** and **B** Patients with positive TS CD68^+^iNOS^+^ M1 TAM showed higher OS (p-value = 0.0585) and RFS (p-value = 0.0303) than those with negative TS CD68^+^iNOS^+^ M1 infiltration; **C** and** D** Patients with positive TN CD68^+^CD206^+^ M2 TAM showed significantly worse OS (p-value < 0.0001) and RFS (p-value < 0.0001) outcome than those with no TN CD68^+^CD206^+^ M2 infiltration; **E** and **F** Patients with high TS CD68^+^CD206^+^ M2 TAM infiltrating level showed worst OS and RFS outcome among all patients, significantly worse than those of no and low TS CD68^+^CD206^+^ M2 infiltration (p-value < 0.0001, respectively); **G** and **H** Validation cohort using maximum IHC section of whole LSCC tumor tissue. **G** Representative CD68 and CD206, CD68 and iNOS IHC staining image in IHC serial section of LSCC tissues with different recurrence-free survival outcomes; **H** Patients who suffered from tumor recurrence within one year showed significantly higher CD68^+^CD206^+^ M2 high-infiltration rate than those who showed no tumor recurrence five years after initial surgery (p-value = 0.035). *OS*, Overall survival; *RFS*, Recurrence-free survival; *TS*, Tumor stroma; *TN*, Tumor nest; *IHC*, Immunohistochemical. OS and RFS rates were calculated according to the Kaplan–Meier method, and statistical differences between the different groups were calculated using the log-rank test

We performed Kaplan–Meier analysis and the log-rank test to evaluate the correlation between iNOS^+^ or CD206^+^ TAMs infiltration and patient survival. Only 1 (3.8%) of the 13 patients with positive TS iNOS^+^ TAMs infiltration exhibited tumor recurrence at 28 months and died at 40.0 months after initial treatment. The analysis showed that positive TS iNOS^+^ TAMs infiltration indicated a significantly better recurrence-free survival (RFS) (p-value = 0.0303) and overall survival (p-value = 0.0585, Fig. 3A, B) compared to those with no TS iNOS^+^ TAMs infiltration. In contrast, patients with positive TN CD206^+^ TAMs infiltration showed significantly worse recurrence and survival outcomes than those with no TN CD206^+^ infiltration (p-value < 0.0001, Fig. 3C, D). We did not find a statistical significant correlation between TS CD206^+^ TAMS and patient RFS or OS. However, a subgroup of patients with high TS CD206 + TAMs infiltration showed markedly worse RFS and OS survival compared to those with low or negative CD206 + TAM infiltration (p-value < 0.0001, Fig. 3E, F).

### Maximum immunohistochemical (IHC) section of whole LSCC tumor tissue in validation cohort confirmed the prognostic value of CD206 + TAMs

To validate the correlation between the infiltration of TAMs subgroups and the prognosis of LSCC patients, we reviewed the whole medical history and pathological section materials of 20 LSCC patients who underwent initial tumor resection without any postoperative adjuvant radiotherapy or chemotherapy at a single clinical center in 2015. Thirteen (65.0%) of the 20 patients showed tumor recurrence within one year after primary surgical treatment, however, the other 7 (35.0%) showed no tumor recurrence over five years after initial surgery, indicating a significantly better survival outcome. Next, we examined the infiltration of TAMs on the pathological section materials of whole tumor tissue using immunohistochemical staining. The result indicated that most patients with no recurrence showed no or low CD206^+^ TAMs infiltration and only 2 (28.6%) patients in this group exhibited high CD206^+^ TAMs infiltrating levels. However, 10 (76.9%) in the 13 patients in the one-year recurrence group showed a high level of CD206^+^ TAMs infiltration. In addition, the rate of high CD206^+^ TAMs infiltration in the one-year recurrence group was significantly higher than that in no recurrence group (p-value = 0.035, Fig. 3G, H). We also tested the infiltration of iNOS^+^ TAMs and found that 4 and 2 patients in the no recurrence group and one-year recurrence group showed positive iNOS^+^ TAMs infiltration, respectively. Consistently, patients with satisfactory long-term recurrence-free survival generally showed positive iNOS^+^ TAMs infiltration (57.1% and 15.4%, respectively, p-value = 0.052, Fig. 3G, H).

### Molecular phenotypic characteristics analysis of iNOS+ TAMs and CD206+ TAMs using flow cytometry

**Fig. 4:**
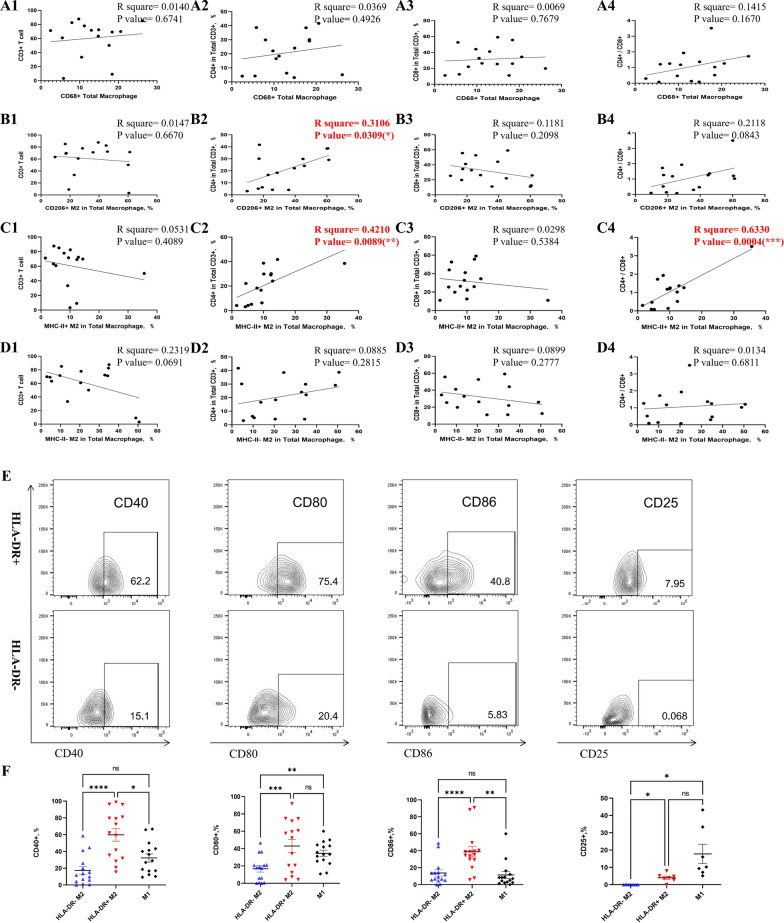
The correlation between tumor-infiltrating T lymphocytes and TAMs of fresh LSCC tissue samples using flow-cytometric analysis (n = 15). **A** The correlation between CD68^+^ total TAM infiltration and total CD3^+^ T cell **A1**, CD4^+^ T cell in total CD3^+^ **A2**, CD8^+^ T cell in total CD3^+^ **A3**, and CD4^+^/CD8^+^ rate **A4**; (B) The correlation between CD68^+^CD206^+^ M2 TAM infiltration and total CD3^+^ T cell **B1**, CD4^+^ T cell in total CD3^+^ **B2**, CD8^+^ T cell in total CD3^+^ **B3**, and CD4^+^/CD8^+^ rate **B4**; **C** The correlation between CD68^+^HLA-DR^high^CD206^+^ M2 subgroup infiltration and total CD3^+^ T cell **C1**, CD4^+^ T cell in total CD3^+^ **C2**, CD8^+^ T cell in total CD3^+^ **C3**, and CD4^+^/CD8^+^ rate **C4**; **D** The correlation between CD68^+^HLA-DR^low/−^CD206^+^ M2 subgroup infiltration and total CD3^+^ T cell **D1**, CD4^+^ T cell in total CD3^+^ **D2**, CD8^+^ T cell in total CD3^+^ **D3**, and CD4^+^/CD8^+^ rate **D4**. The presence of costimulatory molecule for T-cell activation including CD40 (first column), CD80 (second column), CD86 (third column) and CD25 (last column) in CD68^+^HLA-DR^low/−^CD206^+^ M2, CD68^+^HLA-DR^high^CD206^+^ M2, and CD68^+^iNOS^+^ M1 subgroups **E**, and the scatter plot (with mean ± SD) comparing fractions of the above-mentioned surface antigens within the three TAM subsets **F**. *TAMs*, Tumor-associated macrophages. Linear regression analyses were used to test the correlation between different subgroups of cells

Next, we investigated the immune cell composition in the TME in fresh tumor tissue samples to gain insights into how TAMs interact with other cells. Flow cytometry results revealed that the infiltration of total CD68^+^ TAMs was not associated with CD3^+^ tumor-infiltrating T cells (TILs) including CD4^+^ TILs, CD8^+^ TILs, and CD4 + /CD8 + TILs (Fig. 4, panels A1-A4). However, the CD206^+^ TAM subgroup showed strong correlation with CD4^+^ TILs (R square = 0.3007, p-value = 0.0343, Fig. 4, panels B1-B4). Considering the interaction of major histocompatibility complex class II (MHC-II) with CD4^+^ T cell receptors (TCRs) plays a central role in CD4^+^ T cell immunity, we divided CD206^+^ TAMs into HLA-DR^high^ and HLA-DR^low^ subgroups and analyzed the correlation of each with CD4^+^ TILs. Interestingly, HLA-DR^high^ CD206^+^ TAMs showed strong correlation with both CD4^+^ TILs and CD4 + /CD8 + ratio (R square = 0.4044 and 0.6190, p-value = 0.0108 and 0.0005, respectively, Fig. 4, panels C1-C4). In contrast, no correlation was found between HLA-DR^low^ subgroup with CD3 T cell and its subtypes (CD4 + , CD8 + and CD4 + /CD8 + ratio, p-value > 0.05, Fig. 4, panels D1-D4).

To investigate the functional role of HLA-DR^high^ and HLA-DR^low/−^ TAMs in the TME of LSCC, we analyzed the expression of costimulatory molecule for T-cell activation including CD40, CD80 and CD86 in each subset (Fig. 4E). We discovered that HLA-DR^high^ CD206^+^ TAMs showed significantly higher CD40 and CD86 expression than either HLA-DR^low/−^ CD206^+^ (p-value < 0.0001) or iNOS^+^ TAMs (p-value = 0.0257 and 0.0032, respectively) whereas the expression of CD80 of HLA-DR^high^ CD206^+^ TAMs and iNOS^+^ TAMs was similar (p-value = 0.5485), and both were higher than that of HLA-DR^low/−^ TAMs (p-value = 0.0002 and 0.0022, respectively). Interestingly, we also found that CD25^+^ expressing TAMs was nearly absent in HLA-DR^low/−^CD206^+^ TAMs, whereas it exited in HLA-DR^high^ CD206^+^ with a range of 0% to 7.89% and iNOS^+^ TAMs with a range of 5.1% to 43.2%. The difference was statically significant (p-value = 0.0424 and 0.0117, respectively Fig. 4F).

### Co-localization of HLA-DR^high−^CD206^+^ TAMs and CD4^+^ TILs in the tumor microenvironment of LSCC

**Fig. 5:**
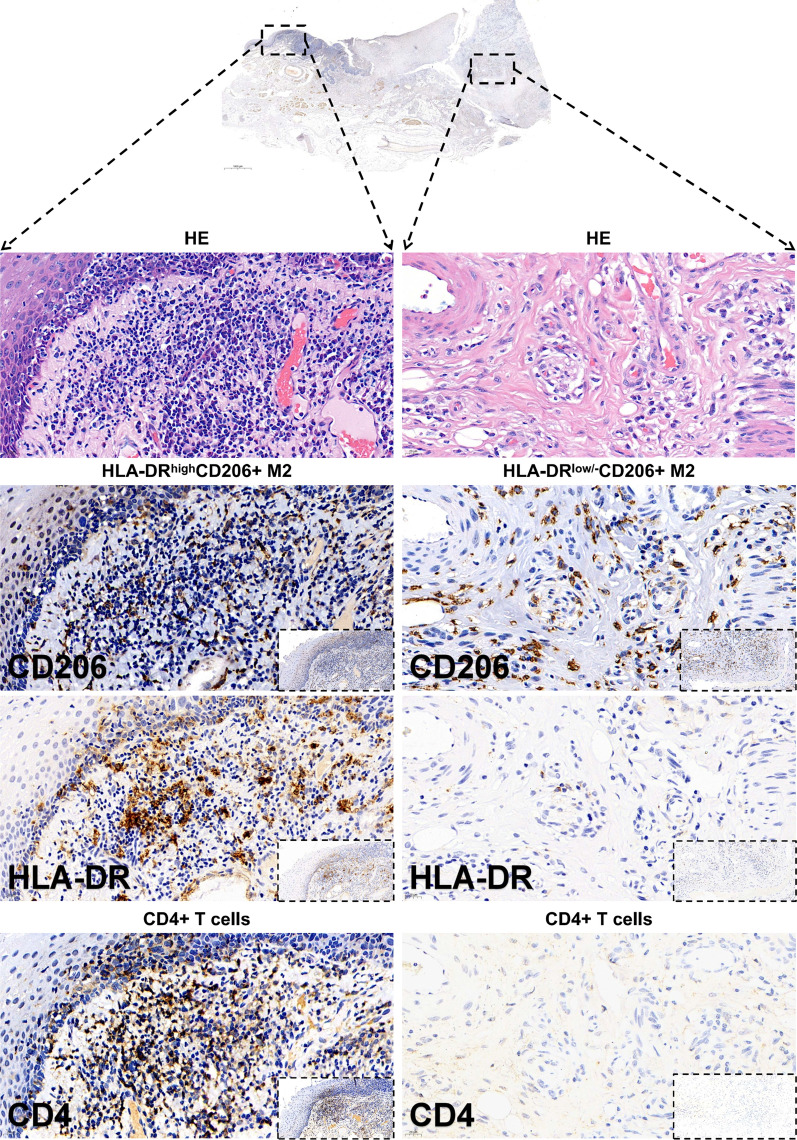
Representative immunohistochemical (IHC) of HLA-DR, CD206 and CD4 using serial LSCC tissue section. Corresponding HE staining and immunohistochemical staining of CD206^+^, HLA-DR^+^ M2-like TAMs and CD4^+^ T lymphocytes were shown as indicated

To further explore the interaction between HLA-DR^high^CD206^+^ TAMs and CD4^+^ TILs in the tumor microenvironment of LSCC, we examined the localization of both types of cells on serial tumor tissue section using immunohistochemical (IHC) staining for HLA-DR, CD206 and CD4. The result showed that in section with high CD206^+^ TAM infiltration was accompanied by the enrichment of CD4 + TILs whereas in area with less CD206 staining, there was also less CD4 + TILs (compare the left and right columns in Fig. 5).

### HLA-DR^high−^CD206^+^ and CD4^+^ TILs co-localize with apoptotic tumor cells in the tumor microenvironment of LSCC

**Fig. 6:**
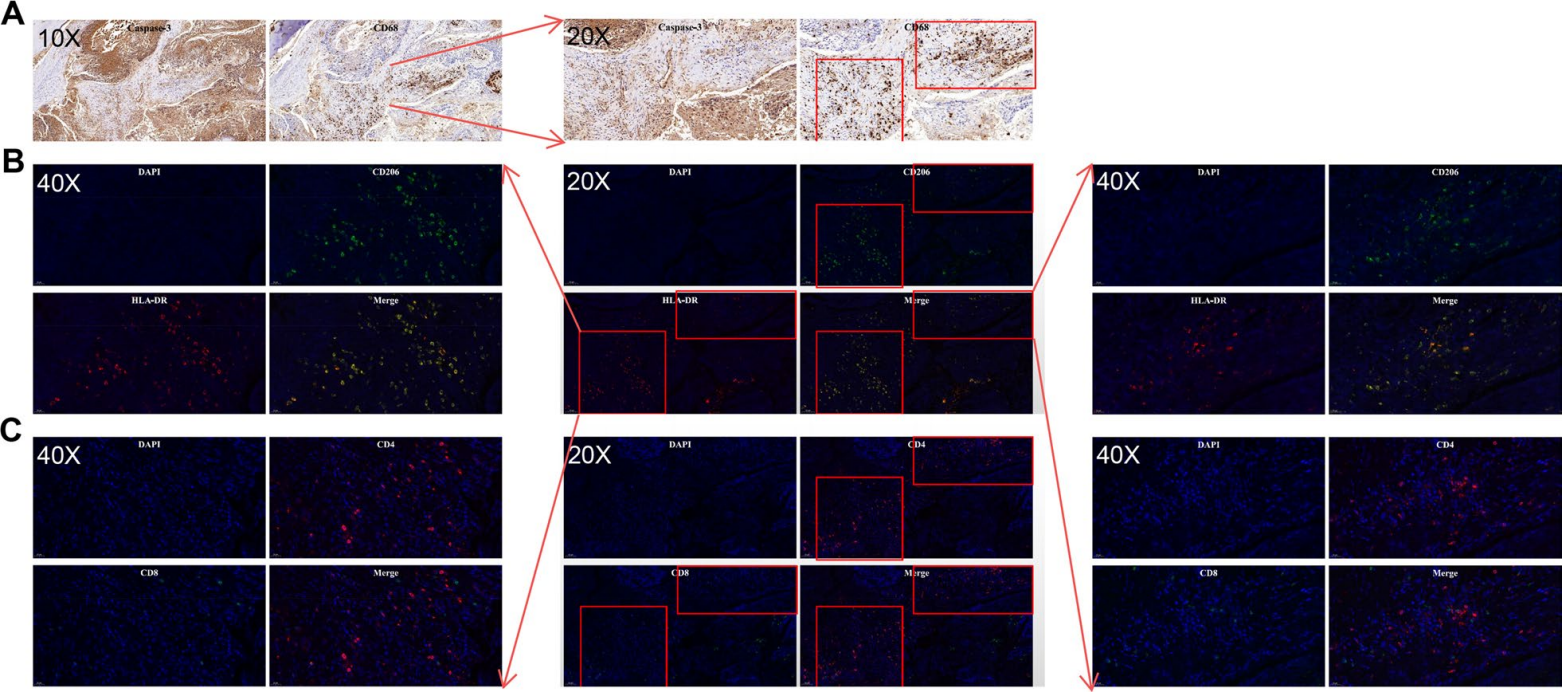
Representative image of co-localization of Cleaved-Caspase 3 + apoptotic tumor cells, HLA-DR^high^CD206^+^ M2 TAMs, and CD4^+^ TILs in the tumor microenvironment of LSCC serial tissue section. **A** Immunohistochemical staining of Cleaved Caspase-3^+^ tumor cell and CD68 + TAMs; **B** Double-labeling immunofluorescence of HLA-DRhighCD206 + M2 TAMs; **C** Double-labeling immunofluorescence of CD4 + and CD8 + TILs using serial sections of LSCC

Given that apoptotic tumor cells regulate the TME, including the modulation of macrophage polarization [21, 22], we used immunohistochemical and double-labeling immunofluorescence staining of serial tumor tissue section to investigate the localization of TAMs. We found that HLA-DR^high^ CD206^+^ macrophages preferentially accumulated around areas with a large number of Cleaved Caspase-3^+^ apoptotic tumor cells (Fig. 6 and Additional file 4: Fig. S4).

## Discussion

Macrophage plays a significant role in innate immune responses against pathogens and is also reported to be one of the main cellular ingredients in the microenvironment of many solid tumors [23]. Tumor-associated macrophages (TAMs), modulated by several signals from the local region of the tumor, can take part in almost all aspects of tumorigenesis [24], and have been proven to be associated with the prognosis of several solid tumors [11–15, 17].

Accumulating evidence indicates that CD68 is highly expressed by human tissue macrophages, and is labeled as a pan-macrophage biomarker [25]. CD68^+^ macrophages have been related to both favorable and unfavorable outcomes in different types of cancer. These ambiguous results may be due to a lack of more nuanced stratification. As a complex community, many external stimuli exist in the tumor microenvironment and may drive TAMs to different phenotypes with opposing properties, [26, 27]. M1 macrophage, which is promoted by Th1-derived cytokines such as interferon-γ (IFN-γ), has been reported to be a resistant factor in tumorigenesis [28], while M2, induced by Th2-derived cytokines such as IL-4 and IL-10, usually display a pro-tumorigenic role [29].

In our research, we demonstrated that identification of intratumoral M1- and M2-like macrophage polarization using iNOS and CD206 and distribution using tumor stroma and nest regions can predict recurrence and survival outcome for laryngeal squamous carcinoma (LSCC) patients who received radical tumor resection. Intratumoral iNOS^+^ M1-like TAMs infiltration was relatively scarce and they mainly localized in the stroma region within the tumor, and hardly any iNOS^+^ TAMs could infiltrate into the tumor nest. The expression of NOS1 by cancer cells has been proven as having immune dysfunction effect by IFN signal dysfunction in circulating immune cells in patients with melanoma [30]. However, the expression of NOS gene by macrophage (M1 subset) usually predict better survival outcome in several cancers [28]. Furthermore, in our current research, the infiltration of iNOS^+^ TAM was a prognostic factor for satisfactory RFS and OS in LSCC patients. On the contrary, CD206^+^ M2-like TAMs infiltration was more common in LSCC patients, and they infiltrated to not only the stroma region but also tumor nest. High infiltrating levels of both tumor nest and stroma CD206^+^ M2 macrophage were associated with poor prognosis in LSCC patients.

CD206, also named the mannose receptor (MR), is one of the C-type lectin superfamily [31]. As a type-I membrane protein, CD206 comprises a single transmembrane domain as well as a cytoplasmic domain which plays an important role in receptor internalization and recycling [32]. CD206 was initially discovered on rabbit alveolar macrophages, and increasing evidence has suggested that CD206 is closely associated with the functional status of macrophages and was also identified as a phenotypic hallmark of mature M2 macrophages [33]. CD163 (hemoglobin-scavenger receptor) is another recognized surface marker for M2 macrophage, and the high infiltration of CD163^+^ M2 macrophage in the tumor microenvironment (TME) has been proven to be associated with tumor progression and poor prognosis in various kinds of solid tumors [34, 35]. As for head and neck cancer, most of the previous research detected M2 macrophage by surface expression of CD68 and CD163. Hu et al. and Matsuoka et al. both found that higher concentrations of CD163^+^ macrophage were related to worse survival outcomes in oral squamous cell carcinoma [36, 37]. Snietura et al. reported that intensive CD163^+^ macrophage infiltration was an adverse prognostic factor for long-term survival in patients with human papillomavirus-negative oropharyngeal squamous cell carcinoma [38]. We also evaluated the CD163 expression by immunofluorescence for patients with LSCC. Surprisingly, almost no CD163^+^ subgroup was found in TME of LSCC patients, indicating the distinctive, CD206^+^ M2-dominated differentiation pattern of macrophage in the development and progression of LSCC.

We also investigated the relationship between TAMs and tumor-infiltrating T lymphocytes on fresh specimens of LSCC using flow cytometry. Although we did not discover any correlation between total CD68^+^ TAMs and total CD3^+^ T cell as well as its CD4^+^ and CD8^+^ subgroups, we found that CD206^+^ TAMs were closely associated with CD4^+^ tumor-infiltrating T lymphocytes. As one of the antigen-presenting cells, macrophages can present peptides through their major histocompatibility complex class I and II (MHC-I and MHC-II) protein complex to CD8^+^ and CD4^+^ T lymphocytes, respectively. After further stratifying CD206^+^ TAMs as HLA-DR^high^ and HLA-DR^low/−^ subgroups, we found a correlation of cell number and colocalization of HLA-DR^high^ CD206^+^ TAMs with CD4^+^ tumor-infiltrating T lymphocytes in LSCC, which strongly suggest that the CD4^+^ T cell/MHC-II axis plays a key role in the cross-talk of tumor-infiltrating macrophage and T lymphocytes in the TME of LSCC. The HLA-DR^high^CD206^+^ macrophage may be the principal subgroup of TAMs that interacts with CD4^+^ T lymphocytes.

M1 macrophages are major MHC-II positive cells and are more active in initiating and promoting immune response [39]. However, due to the immunoexpressed TME, polarization toward the pro-tumor M2 phenotype may be favored. Interestingly, HLA-DR^high^ CD206^+^ TAMs showed a higher surface CD86 expression than both HLA-DR^low/−^CD206^+^ and iNOS^+^ M1-like TAMs. For another costimulatory molecule CD80, HLA-DR^high^ subgroup exhibited comparable expression levels with M1-like TAMs, both significantly higher than that of HLA-DR^low/−^ M2-like subgroup. The TNF receptor superfamily member CD40, which can be expressed on macrophage and interacts with its binding partner CD40L which predominantly expresses on activated CD4^+^ T lymphocyte [40], was also presented significantly more frequently on HLA-DR^high^ TAMs compared to HLA-DR^low/−^ and iNOS^+^ TAMs, which once more underlying that this subgroup was the major TAM population to interact with CD4^+^ T cells.

The results indicated that although the function of M1 subgroup was suppressed, there is a group of highly-activated M2 macrophage in the TME of LSCC. M1 and M2 characteristics are not necessarily mutually exclusive, and often co-exist. Unlike the traditional activation pathway of M1 macrophage which was mainly mediated by IFN-γin TME of LSCC [41], the activation level of M2 TAM depended mostly on the complex immune microenvironment of tumor, especially the co-reaction of CD4^+^ T cell/MHC-II axis. Furthermore, mediated by activated M2 cells, CD4 T cells tend to differentiate toward Th2 type 2 helper T cells, releasing TH2 cytokines that in turn facilitates macrophage polarization to M2. Through this pathway, a local immunosuppressive microenvironment is established that promotes tumor progression in laryngeal cancer. What’s more, among the TME of LSCC, iNOS^+^ M1 and HLA-DR^high^CD206^+^ M2 TAMs were proven to have comparable elevated expression of CD25 (a surface marker that is more commonly found on mature lymphocytes), which is barely expressed by HLA-DR^low/−^CD206^+^ M2 subgroup.

By analyzing the immunohistochemical and double-labeling immunofluorescence staining results, we also found that the HLA-DR^high^ CD206^+^ TAMs had a distinct tendency to cluster near tumor apoptosis foci within the tumor microenvironment of LSCC. Previous articles have reported that apoptotic colorectal cancer cells release chemotactic factors that induce anti-inflammatory macrophage polarization [22]. Here in our research, the majority of macrophages around the Cleaved Caspase-3^+^ apoptotic tumor cells were of M2 subset.

## Conclusions

This study demonstrated the dominant CD206 + macrophage infiltration pattern in the TME of LSCC. We also proved that CD206 + macrophage infiltration was an independent predictor of poor survival in LSCC patients. Furthermore, we identified a highly activated CD206 + subgroup with elevated expression of the surface HLA-DR, which may be induced by the interaction of CD4 + T cells with MHC-II axis. This may be a critical mechanism in the formation of immunosuppressive microenvironment and tumor progression, resulting in the poor prognosis of LSCC patients.

## Supplementary Information

**Additional file 1: Figure S1.** The division of tumor regions (tumor nest region (red) and tumor stroma region (green)) in the IHC (A) and immunofluorescence (B,C) staining.

**Additional file 2: Figure S2.** Gating strategy of TAMs and TILs for flow cytometry. *TAMs*, tumor-associated macrophages; *TILs*, tumor-infiltrating T lymphocytes.

**Additional file 3: Figure S3.** Representative multiplex immunofluorescence staining of CD68 (B), CD206 (C), and iNOS (D) of LSCC tissue.

**Additional file 4: Figure S4.** Co-location of Cleaved-Caspase 3+ apoptotic tumor cells, HLA-DR^high^CD206^+^ M2 TAMs, and CD4^+^ TILs in the tumor microenvironment of LSCC using serial tissue section.

**Additional file 5: Table S1.** Data of antibodies used in our research.

## Data Availability

The datasets used and/or analysed during the current study are available from the corresponding author on reasonable request.

## Abbreviations

TAM: Tumor-associated macrophage
HE: Hematoxylin and eosin
TS: Tumor stroma
TN: Tumor nest
LSCC: Laryngeal squamous cell carcinoma
TME: Tumor microenvironment
TMA: Tissue microarray
TILs: Tumor-infiltrating T cells
MHC-I: Major histocompatibility complex class I
MHC-II: Major histocompatibility complex class II
TCRs: T cell receptors
