# Supplementary figures and images for: CD206+ tumor-associated macrophages interact with CD4+ tumor-infiltrating lymphocytes and predict adverse patient outcome in human laryngeal squamous cell carcinoma
Source: J Transl Med. 2023 Mar 3;21:167. doi: 10.1186/s12967-023-03910-4 (PMC9983170; doi:10.1186/s12967-023-03910-4)

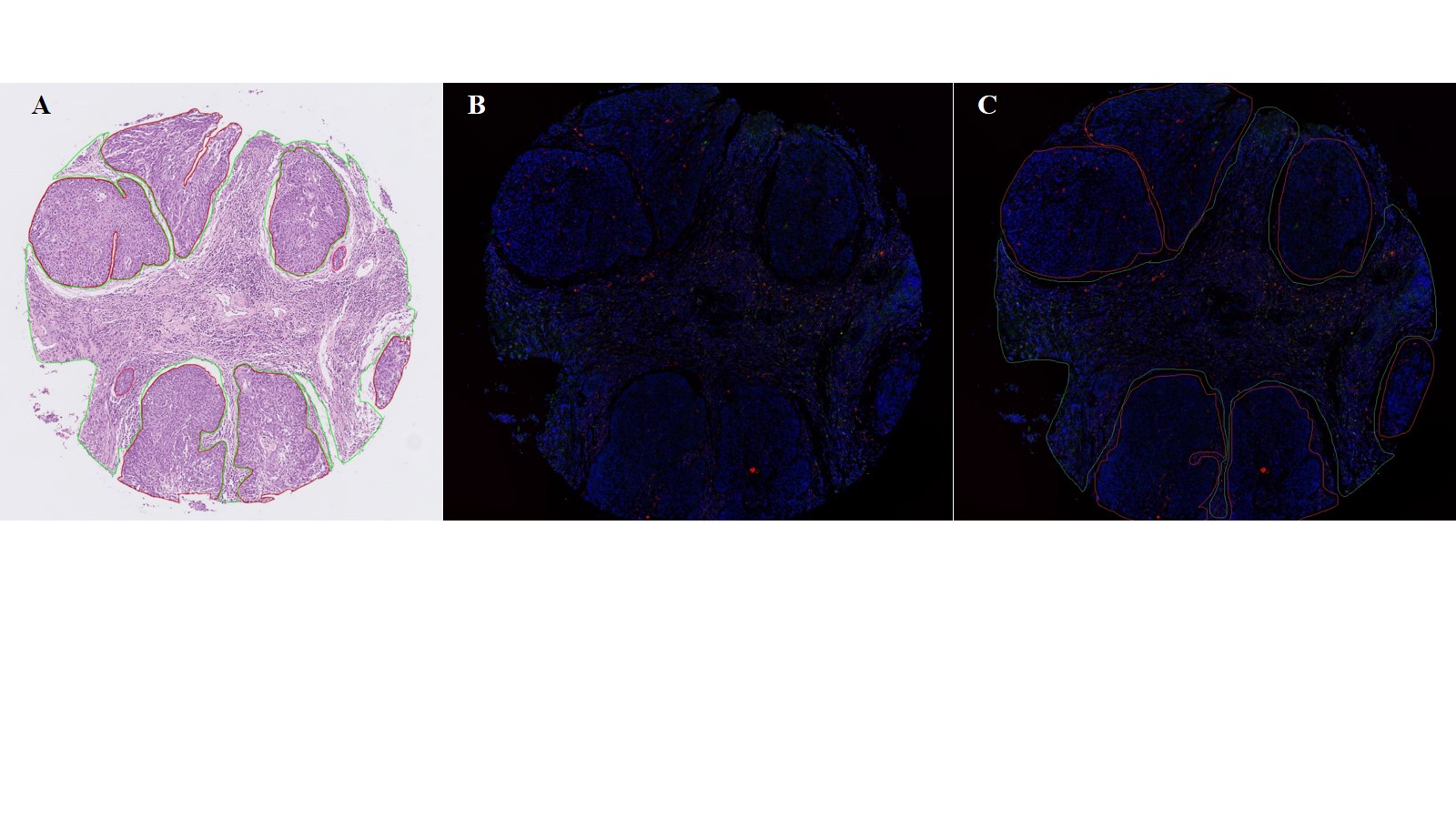

Supplement: Supplementary file 1 — Additional file 1: Figure S1. The division of tumor regions (tumor nest region (red) and tumor stroma region (green)) in the IHC (A) and immunofluorescence (B,C) staining. [file 12967_2023_3910_MOESM1_ESM.jpg]

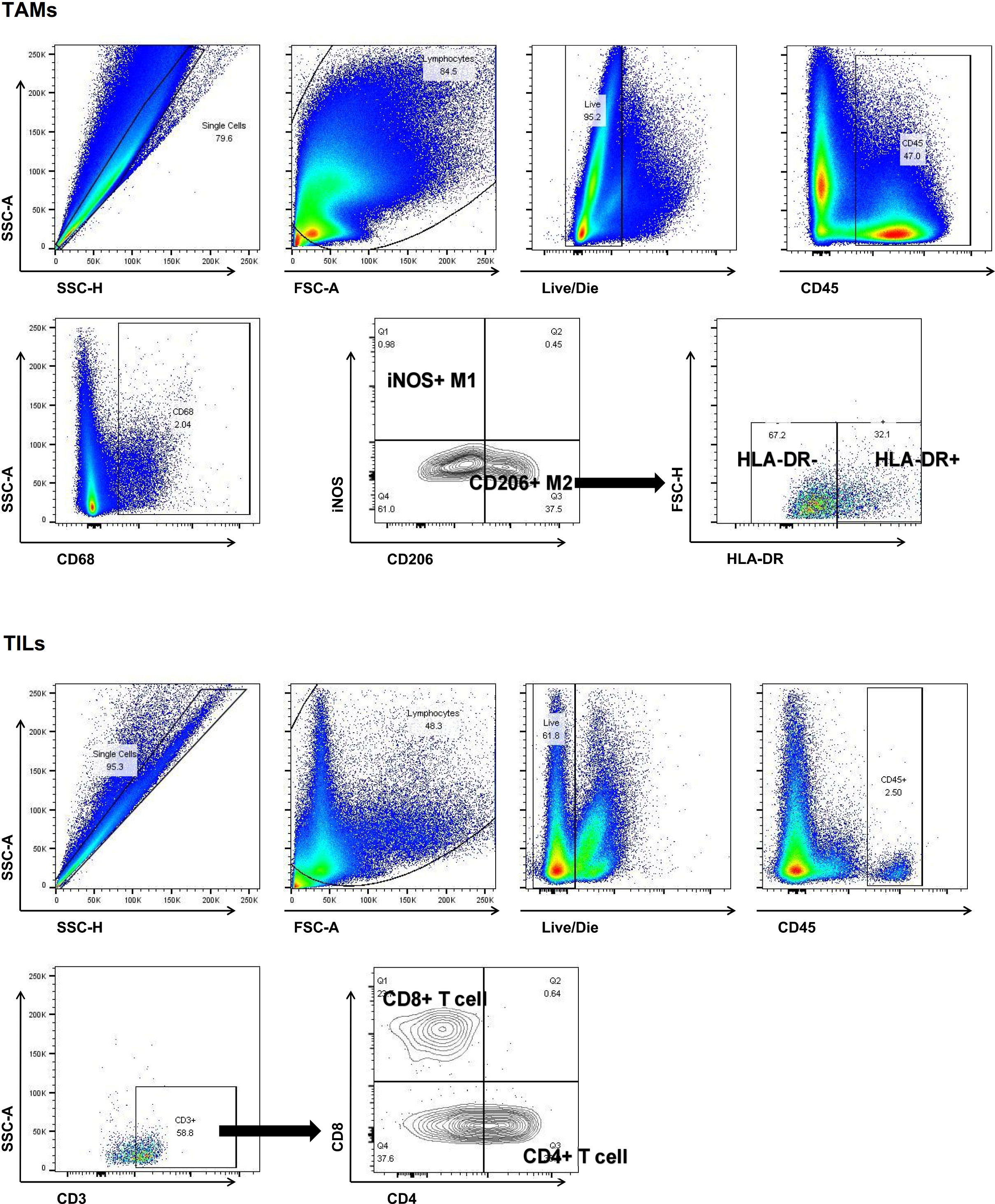

Supplement: Supplementary file 2 — Additional file 2: Figure S2. Gating strategy of TAMs and TILs for flow cytometry. TAMs, tumor-associated macrophages; TILs, tumor-infiltrating T lymphocytes. [file 12967_2023_3910_MOESM2_ESM.tif]

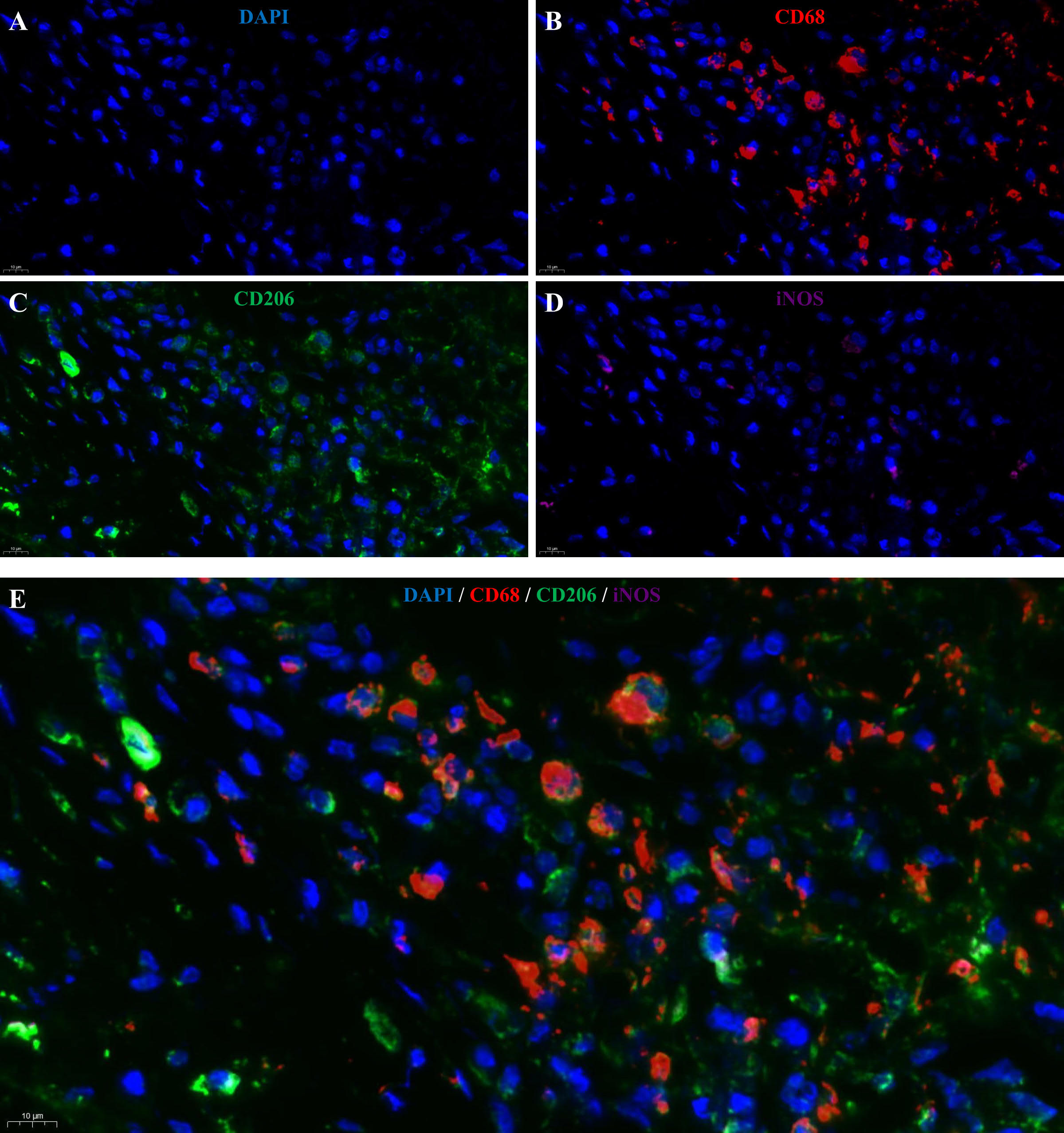

Supplement: Supplementary file 3 — Additional file 3: Figure S3. Representative multiplex immunofluorescence staining of CD68 (B), CD206 (C), and iNOS (D) of LSCC tissue. [file 12967_2023_3910_MOESM3_ESM.jpg]

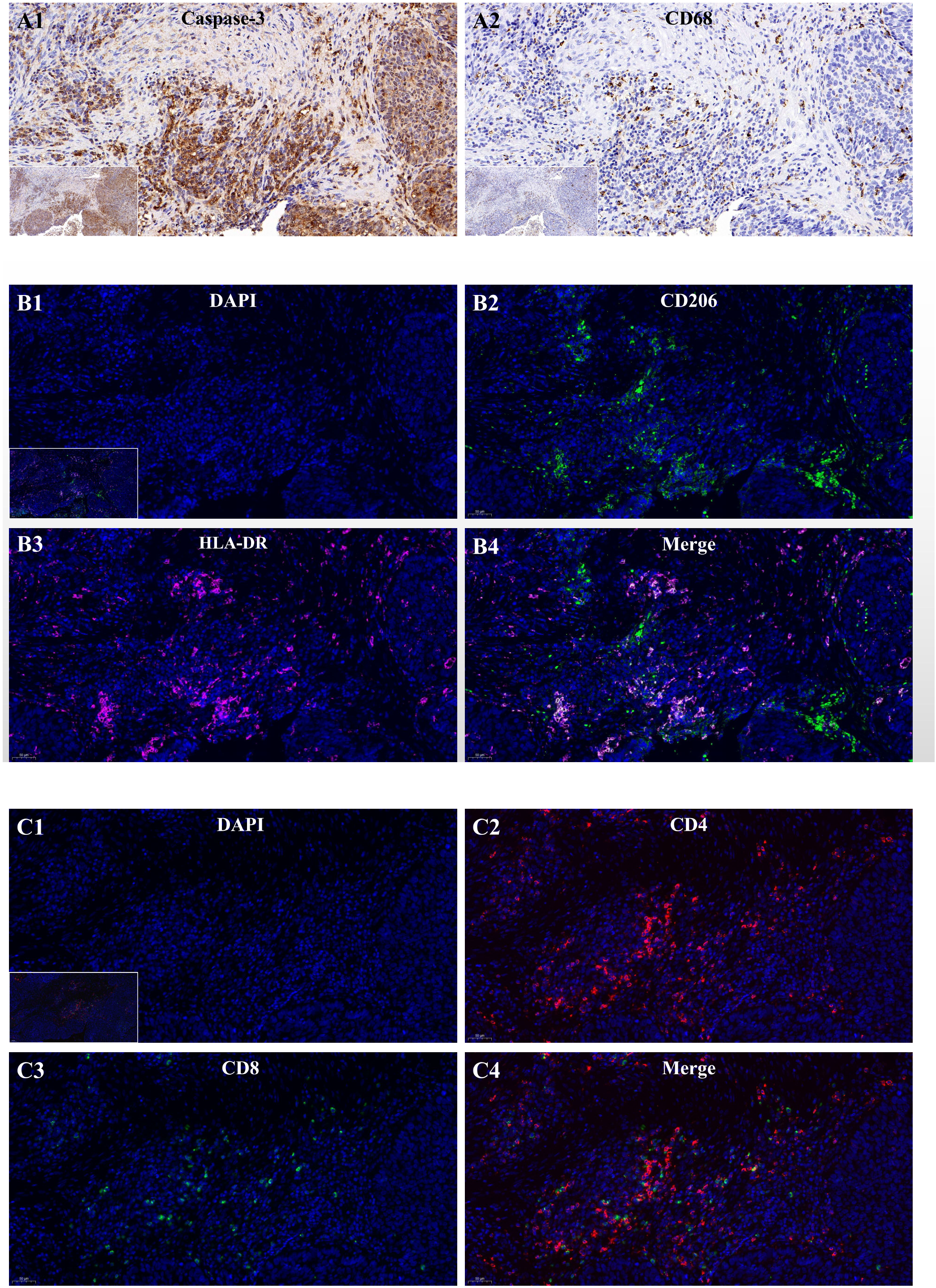

Supplement: Supplementary file 4 — Additional file 4: Figure S4. Co-location of Cleaved-Caspase 3+ apoptotic tumor cells, HLA-DRhighCD206+ M2 TAMs, and CD4+ TILs in the tumor microenvironment of LSCC using serial tissue section. [file 12967_2023_3910_MOESM4_ESM.tif]
