# Supplementary material for: CD206+ tumor-associated macrophages interact with CD4+ tumor-infiltrating lymphocytes and predict adverse patient outcome in human laryngeal squamous cell carcinoma
Source: J Transl Med. 2023 Mar 3;21:167. doi: 10.1186/s12967-023-03910-4 (PMC9983170; doi:10.1186/s12967-023-03910-4)
Supplement: Supplementary file 5 — Additional file 5: Table S1. Data of antibodies used in our research. [file 12967_2023_3910_MOESM5_ESM.docx]

**Additional file Table S1:** Data of antibodies used in our research

| **Antibody** | **Cat#** | **Fluorochrome** | **Company** |
| --- | --- | --- | --- |
| CD45 | 304050 | Brilliant Violet 711 | BioLegend |
| CD68 | 564943 | Brilliant Violet 421 | BD Biosciences |
| CD206 | 321120 | APC/Cyanine7 | BioLegend |
| iNOS | 25-5920-82 | PE-Cyanine7 | eBioscience |
| HLA-DR | 327020 | PerCP/Cyanine5.5 | BioLegend |
| CD3 | 300428 | PerCP | BioLegend |
| CD4 | 317418 | APC/Cyanine7 | BioLegend |
| CD8 | 300906 | FITC | BioLegend |
| CD80 | 305208 | PE | BioLegend |
| CD86 | 305412 | APC | BioLegend |
| CD40 | 334336 | Brilliant Violet 421 | BioLegend |
| CD25 | 302604 | FITC | BioLegend |
